# Si xian formula (SXF) alleviates carboplatin-induced bone marrow microenvironment damage and promotes thrombocytopenia recovery by regulating gut microbiota and bone marrow metabolites: a correlative study

**DOI:** 10.3389/fphar.2026.1689477

**Published:** 2026-04-10

**Authors:** Fang Li, Meng-yao Yu, Nan Jiang, Mei-yi Yao, Xiao-yan Xu, Li-ming He, Zhou zhou, Xia Luo

**Affiliations:** 1 Sichuan Academy of Chinese Medicine Sciences, Fungal Medicine Institute, Chengdu, Sichuan, China; 2 Quality Research and Drug Innovation of Traditional Chinese Medicine Key Laboratory of Sichuan Province, Chengdu, Sichuan, China

**Keywords:** bone marrow microenvironment, chemotherapy-induced thrombocytopenia, gut microbiota, metabolites, si xian formula

## Abstract

**Introduction:**

Chemotherapy-induced thrombocytopenia (CIT) presents as a prevalent hematological toxicity, often complicating the clinical management of cancer patients. SI XIAN Formula (SXF), a prescription consisting of ten traditional Chinese medicine for treating chemotherapy-induced thrombocytopenia (CIT), has been widely used to treat CIT patients more than 10 years. The mechanism of SXF remains unclear. This study was designed to systematically evaluate the therapeutic efficacy and elucidate the underlying mechanisms of SXF in a CIT mouse model.

**Methods:**

A CIT mice model was successfully established via the tail vein injection of carboplatin. The Mice were subsequently treated daily with SXF for 15 days and 24 days. Body weight and platelet count were recorded every 3 days. The bone marrow cells and fluid were collected for flow cytometry analysis and cytokine detection separately. Histological changes in the femurs bone marrow were observed using H&E staining. The diversity and composition of the intestinal microbiota were analyzed using 16S rDNA gene sequencing. The bone marrow metabolites were identified by HPLC-MS/MS analysis.

**Results:**

SXF administration significantly accelerated the recovery of platelet counts to baseline levels following carboplatin -induced thrombocytopenia. Mechanistically, SXF demonstrated protective effects on multiple hematopoietic cells, including hematopoietic stem/progenitor cells, megakaryocytic progenitor cells, and bone marrow megakaryocytes in CIT-induced mice. Furthermore, SXF effectively restored carboplatin-induced alterations in hematopoietic cytokine profiles. SXF treatment altered gut microbial diversity and community structure, particularly reducing the abundance of *Paraprevotella* and *Bacteroides* while increasing the abundance of *Lachnospiraceae_NK4A136* compared with the model group, which is positively correlated with all hematopoietic function indicators (P < 0.001). Metabolomic profiling further revealed that SXF ameliorated the bone marrow metabolic profile in CIT-induced mice. Spearman correlation analysis revealed significant positive associations between *Parabacteroides*, *Prevotellaceae_UCG-001*, *Bacillu*s, and unclassified *Muribaculaceae* with upregulated metabolites, while showing inverse correlations with downregulated metabolites in SXF(H) versus MDL group comparisons. Our findings demonstrate that SXF alleviates CIT in mice by orchestrating a novel gut-bone marrow regulatory axis, bridging gut microbiota and marrow metabolic remodeling.

**Conclusion:**

In CIT murine models, SXF administration was shown to alleviate carboplatin-induced bone marrow microenvironment injury and accelerate platelet recovery via bidirectional regulation of gut microbiota and marrow metabolic remodeling.

## 1 Introduction

Chemotherapy-induced thrombocytopenia (CIT) is one of the most common hematological toxic reactions during anthracycline, platinum, paclitaxel or gemcitabine-based chemotherapy in cancer patients with solid tumors, with the incidence of 10%–38% (Shaw et al., 2021; Weycker et al., 2019; Wu et al., 2022), among which the incidence of thrombocytopenia caused by carboplatin can even reach 81.8% (Ten Berg et al., 2011). CIT patients present with platelet counts below 100 × 10^9^/L, manifesting as cutaneous or mucosal bleeding in mild cases, while severe cases face increased risks of life-threatening hemorrhage, compromised treatment efficacy, and escalated medical costs (Danese et al., 2020). CIT may also lead to a reduction in the dosage of chemotherapy drugs, a delay in the chemotherapy schedule, or even the termination of chemotherapy for patients (Kuter, 2022; Mones and Soff, 2019). The main treatments for CIT include platelet transfusion for platelet count below 10 × 10^9^/L (Schiffer et al., 2018) and the application of thrombopoietic drugs including rhTPO and rhIL-11. However, clinical reports indicate that these conventional therapies may induce adverse reactions such as edema, arrhythmia, and myalgia (Xu et al., 2018; Zhang et al., 2022). Therefore, new treatment strategies for CIT need to be explored.

Platelets, produced by bone marrow megakaryocytes, are released into the bloodstream to maintain hemostasis. In the bone marrow microenvironment, megakaryocytes undergo differentiation from hematopoietic stem cells through a tightly regulated process orchestrated by multiple cytokines and signaling pathways, which collectively govern their development, maturation, and platelet-producing capacity (Comazzetto et al., 2021; Lucas, 2021; Pinho and Frenette, 2019). Chemotherapeutic drugs with hematotoxicity damage the hematopoietic microenvironment of bone marrow, leading to the aging of hematopoietic stem cells, impairing proliferation, differentiation and maturation ability of megakaryocytes, and lowered platelet count for a long time (Greenberg, 2017; Kuter, 2015).

Emerging evidence demonstrates that the gut microbiome, a vital constituent of the human microbial ecosystem, exhibits significant associations with bone marrow hematopoietic activity, contributing substantially to hematopoietic homeostasis and modulating the pathogenesis of hematopoietic disorders (Liu et al., 2024; Wells et al., 2024). Disruptions in the compositional and functional profile of intestinal microorganisms significantly modulate both the pathogenesis and treatment responsiveness of diverse hematopoietic diseases (Adeniyi et al., 2023; Cao et al., 2023; Zheng et al., 2024). Moreover, the gut microbiota can affect the functions of hematopoietic stem cells (HSCs) through multiple pathways, including regulating the self-renewal and differentiation of HSCs and the hematopoietic microenvironment (Jiang et al., 2024; Wang et al., 2019). Additionally, metabolic alterations in cells and organisms can induce epigenetic changes in the bone marrow, thereby affecting hematopoietic stem cell (HSC) function (Zhang et al., 2023).

Si xian formula (SXF) was originally developed by Professor Xie Gang, a renowned TCM expert in Sichuan Province, with over 10 years of clinical application history specifically for CIT. Clinical protocols recommend monotherapy with SXF when platelet counts range from 50 × 10^9^/L to 100 × 10^9^/L. For moderate thrombocytopenia cases with platelet counts between 20 × 10^9^/L and 50 × 10^9^/L, SXF is typically combined with either recombinant human interleukin-11 (rhIL-11) or recombinant human thrombopoietin (rhTPO). In cases of severe thrombocytopenia, characterized by platelet counts below 20 × 10^9^/L, SXF administration is accompanied by platelet transfusion therapy.

**FIGURE 1:**
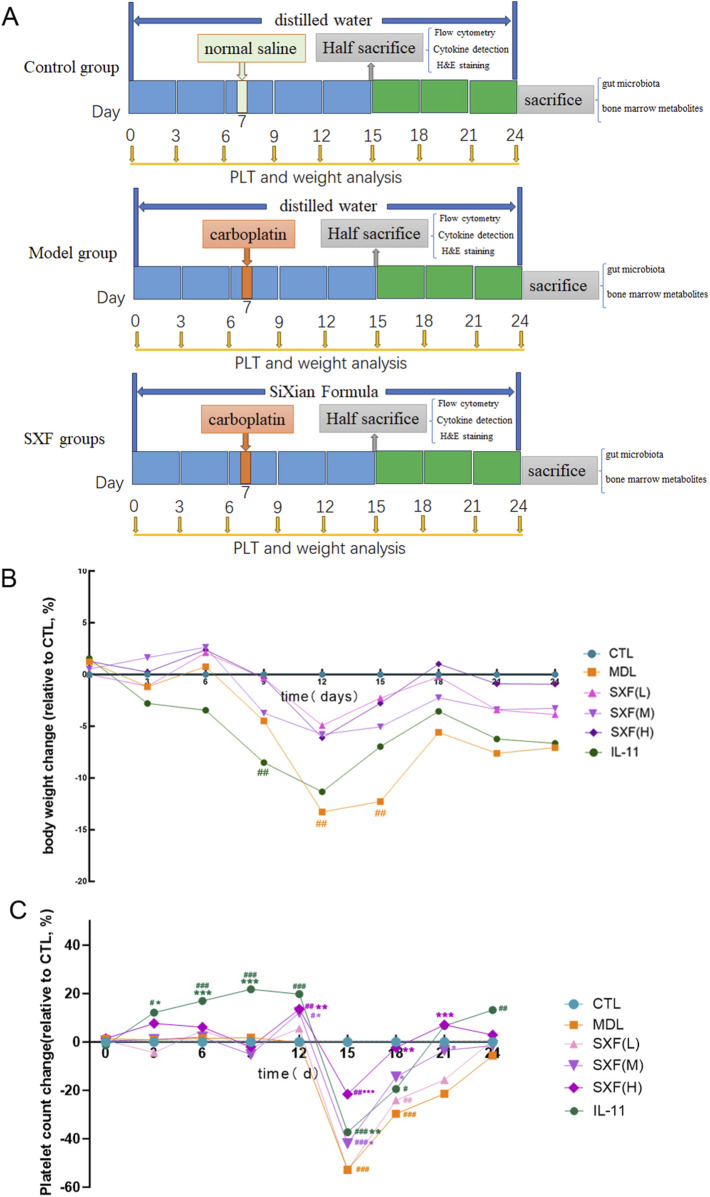
Therapeutic effects of SXF on body weight and Platelet count test. **(A)** Schematic of experimental design. **(B)** Body weights of mice during the 24-days drugs administration. **(C)** Platelet counts of mice during the 24-days drugs administration. Data are reported as the mean ± SD (n = 10). ^###^p < 0.001, ^##^p < 0.01, ^#^p < 0.05 versus the CTL group. ***p < 0.001, **p < 0.01, *p < 0.05 versus the MDL group.

The SXF formulation comprises precisely 10 botanical drugs, as follows: *Curculigo orchioides* Gaertn. [Hypoxidaceae; *C. orchioides* rhizome], *Epimedium brevicomu* Maxim [Berberidaceae; *E. brevicomu* folium], *Agrimonia pilosa* Ledeb [Rosaceae; *A. pilosa* herba], *Ganoderma lucidum* (Leyss. ex Fr.) Karst. [Polyporaceae; *G. lucidum* fruit body], *Poria cocos* (Schw.) Wolf [Polyporaceae; *P. cocos* sclerotium], *Astragalus membranaceus* (Fisch.) Bge. [Fabaceae; *A. membranaceus* radix], *Spatholobus suberectus* Dunn [Fabaceae; *S. suberectus* caulis], *Curcuma xvenyujin* Y. H. Chen et C. Ling [Zingiberaceae; *C. xvenyujin* radix], *Forsythia suspensa* (Thunb.) Vahl [Oleaceae; *F. suspensa* fructus], *Ziziphus jujuba* Mill [Rhamnaceae; *Z. jujuba* fructu]. Their quantitative formulation parameters are clearly specified in Table 1. Among these, *C. orchioides* (Jiuxianmao), *E. brevicomu* (Yinyanghuo), *A. pilosa* (Xianhecao), and *G. lucidum* (Lingzhi) have been revered as “celestial botanical drug(s)” (Xian Pin) in traditional Chinese medicine since ancient times, primarily employed for replenishing qi (vital energy) and nourishing blood. Modern pharmacological research has demonstrated that *E. brevicomu* (Yinyanghuo) contains active metabolites such as icariin, exhibiting anti-inflammatory, immunomodulatory, antitumor, and cardioprotective effects, rendering it applicable in cancer therapy (Ding et al., 2024; Luo et al., 2024; Bi et al., 2022). Similarly, *A. membranaceus* contains bioactive metabolites such as astragaloside IV, demonstrating immunomodulatory and antitumor activities, with promising therapeutic potential in mitigating chemotherapy-induced adverse effects (Liang et al., 2023). *Curculigo orchioides* (Jiuxianmao) and *G. lucidum* (Lingzhi) demonstrates therapeutic potential in reversing chemotherapy-induced hematotoxicity and hepatotoxicity (Murali and Kuttan, 2016; Chen et al., 2019; Suárez-Arroyo et al., 2022; Cizmarikova M., 2017). *Ziziphus jujuba Mill* (Dazao) exhibits immunostimulatory and bone marrow cell proliferation-promoting effects (Feng et al., 2025). Additionally, a retrospective clinical study on anemia identified *A. membranaceus* (Huangqi) and *A. pilosa* (Xianhecao) as the most frequently prescribed herbal medicines for stimulating hematopoietic function (Chiu et al., 2021). Therefore, SXF represents a highly promising therapeutic formulation for chemotherapy-induced thrombocytopenia (CIT). A randomized controlled trial (N = 65, stratified by baseline platelet count), oral administration of SXF demonstrated superior clinical response rates versus conventional treatment (primary endpoint: platelet recovery ≥ 100 × 10^9^/L by day 14, 73.7% vs. 37.5%, p = 0.0226) (Tang et al., 2024). The present study was designed to elucidate the therapeutic efficacy and underlying mechanisms of SXF in murine models of chemotherapy-induced thrombocytopenia. The experimental design is shown in Figure 1A.

**TABLE 1:** Herb composition of SXF.

Latin name	Chinese name	Plant part	Batch number	Composition (%)
*Curculigo orchioides* Gaertn	Jiuxianmao	Rhizome	1911006	7.6
*Epimedium brevicomu* Maxim	Yinyanghuo	Folium	1909122	7.6
*Agrimonia pilosa* Ledeb.	Xianhecao	HERBA	191009	9.0
*Ganoderma lucidum* (Leyss. ex Fr.) Karst.	Lingzhi	Fruit body	190923	7.6
*Poria* cocos (Schw.) Wolf	Fuling	Sclerotium	1910084	11.4
*Astragalus membranaceus* (Fisch.) Bge. var. *mongholicus* (Bge.) Hsiao	Huangqi	RADIX	191,118	18.8
*Spatholobus suberectus* Dunn	Jixueteng	CAULIS	1910066	11.4
*Curcuma xvenyujin Y. H. Chen et C. Ling*	Yujin	RADIX	1910027	7.6
*Forsythia suspensa (Thunb.) Vahl*	Lian qiao	FRUCTUS	1911017	11.4
*Ziziphus jujuba Mill*	Dazao	FRUCTUS	1910079	7.6

## 2 Materials and methods

### 2.1 Preparation of SXF

All materials were purchased from licensed vendors and subjected to rigorous quality testing. All herbal materials were quality-tested and certified by Professor Xu Xiaoyan in strict compliance with the Pharmacopoeia of the People’s Republic of China 2020 standards (Chinese Pharmacopoeia Commission, 2020). All voucher specimens were deposited at the Sichuan Academy of Chinese Medicine Sciences. To prepare the alcohol extract of SXF (SXFA), the raw herbal materials of *C. orchioides*, *Epimedium brevicornu*, *A. pilosa*, *S. suberectus*, *Curcuma aromatica*, and *F. suspensa* was extracted twice with 8-fold medicinal ethanol (v/w) at 78 °C for 1 h. The decoction is filtered through a 200-mesh sieve, and the filtrates are combined. The combined filtrate is concentrated under reduced pressure at 55 °C–65 °C to SXFA extract with a relative density of 1.1 g/mL (determined at 60 °C). To prepare the water extract of SXF (SXFW), the raw herbal materials of *A. membranaceus*, *G. lucidum*, *P. cocos*, and *Ziziphus jujube* was extracted twice with 8-fold water (v/w) at 100 °C for 1 h. The decoction is centrifuged at 1,500 r/min, and the centrifugates are combined. The combined solution is concentrated under reduced pressure at 55 °C–65 °C to SXFW with a relative density of 1.1 g/mL (determined at 60 °C). The SXF combined extracts of SXFA and SXFW were concentrated under vacuum evaporation and spray drying. The spraying frequency was controlled at 0.9–1.2 Hz, and the liquid extract was sprayed into the substrate for granulation with the material temperature maintained at 40 °C–60 °C. The extract processing yielded 1 g of concentrated SXF extract from 6.57 g of raw herbal materials, representing an extraction ratio of 1:6.57 (extract to raw material, w/w). The contents of icariin and astragaloside IV in SXF extract were quantified as 39.27 mg and 6.84 mg per 100 g of the herbal materials by HPLC, respectively. Three different chromatograms are shown in Supplementary Figures S1–S3.

### 2.2 Reagents and antibodies

All reagents and antibodies used in this study were sourced from reputable commercial suppliers. Carboplatin was purchased from Qilu Pharmaceutical Co., Ltd. (Hainan, China). Recombinant Human Interleukin-11 for Injection was purchased from Qilu Pharmaceutical Co., Ltd. (Hainan, China). Fluorescein-conjugated monoclonal antibodies against CD34, CD41 a, CD61 were the products of Thermo Fisher Scientific Inc. (Shanghai, China). Mouse PDGFB, TPO, IL-11 elisa Kits and ABplex Mouse 3-Plex Custom Panel was purchased from ABclonal Biotechnology Co., Ltd.

### 2.3 Animals

0ne hundred-twenty healthy specific pathogen-free adult Kunming mice (50% of each sex) weighing 18–22 g were purchased from Sibeifu (Beijing) Biotechnology Co., Ltd., with certificate number SCXK (Jing)2019-0010. Following arrival, animals were acclimatized in the Laboratory Animal Center at Sichuan Academy of Chinese Medicine Sciences, maintained at 18 °C–22 °C with a 12 h photoperiod and free access to food and water. The study was conducted ethically, with animal protocols approved by the Sichuan Academy of Chinese Medicine Sciences (No. R20231019-1).

### 2.4 Group administration and model establishment

The mice were randomly divided into the following six groups (n = 20 in each group): Control group (CTL, distilled water), Model group (MDL, distilled water), SXF low-dose group (SXF (L): 0.375 g/kg), SXF middle-dose group (SXF (M): 0.75 g/kg), SXF high-dose group (SXF (H): 1.5 g/kg), according to 1 mL/100 g gavage and IL-11 group (0.25 mg/kg), according to 1 mL/100 g subcutaneous injection for 14 days. SXF was dissolved in distilled water to prepare three experimental solutions at distinct concentrations for administration. The concentrations of the SXF low-dose group (SXF (L)), SXF middle-dose group (SXF (M)), and SXF high-dose group (SXF (H)) were 0.0375 g/mL, 0.075 g/mL, and 0.15 g/mL, respectively.

Intravenous carboplatin administration (75 mg/kg dose) on treatment day 8 effectively generated a thrombocytopenic mouse model across all test cohorts, with the exception of CTL group. The injection volume was 0.75 mL/100 g body weight. Control group mice received isovolumetric normal saline injections via the tail vein.

### 2.5 Measurement of platelet counts and weights

On day 0, 3, 6, 9, 12, 15, 18, 21, 24, body weights and platelet counts of the mice were recorded. Platelet counts were determined in blood collected from the retro-orbital venous plexus using a species-specific automated hematology analyzer (SYSMEX XT-2000i, Japan).

### 2.6 Flow cytometry analysis for hematopoietic stem cells and megakaryocytes in bone marrow

After 15 days of treatment and final blood sampling, a subset of mice (n = 50%) were humanely euthanized. The right femoral bone marrow was then collected through medium-assisted flushing (RPMI-1640). BM cells were collected to prepare into single cell suspension. After adding fluorescent antibodies targeting CD34-FITC, CD41a-PE and CD61-APC, cell suspensions were incubated on ice for 60 min in darkness. After that, the samples were resuspended in 1 mL PBS for analysis by the BD Accuri C6 flow cytometer.

### 2.7 Cytokine detection

Bone marrow levels of TPO, IL-11, and PDGFB were quantified using commercial ELISA kits according to the manufacturer’s protocols. The cytokine levels of IL-1beta, IL-3 and IL-6 were determined by Mouse 3-Plex Custom Panel based on the manufacturer’s instructions.

### 2.8 Hematoxylin and eosin (H&E) staining

After the blood collection, the mice were sacrificed and left femurs were collected. Femurs were fixed with 4% paraformaldehyde for 3 days. Then the femur samples were embedded in paraffin, sectioned, heated, and dewaxed to water. Femur sections were stained with Hematoxylin and Eosin (H&E) to observe the hematopoietic cells especially megakaryocyte.

### 2.9 16S rDNA amplicon sequencing of feces microbiota

On day 24, put the mice to be sampled into a clean cage lined with sterile filter paper. Immediately collect fecal samples after the mice defecate. Three or four capsules of feces were extracted and stored in a sterilized centrifuge tube (−80 °C). Three cases were randomly sampled as research objects from each group.

Total microbial genomic DNA was extracted from fece samples using the FastPure Stool DNA Isolation Kit (MJYH, shanghai, China) according to manufacturer’s instructions. Prior to analysis, DNA integrity was assessed via 1.0% agarose gel electrophoresis, with subsequent quantification performed by spectrophotometry (NanoDrop® ND-2000) to ensure sample quality (Thermo Scientific Inc., USA) and kept at −80 °C prior to further use. The hypervariable region V3-V4 of the bacterial 16S rRNA gene were amplified with primer pairs 338F (5′-ACT​CCT​ACG​GGA​GGC​AGC​AG-3′) and 806R (5′-GGACTACHVGGGTWTCTAAT-3′) by an T100 Thermal Cycler (BIO-RAD, USA) (Liu et al., 2016). The 20 μL PCR system contained: 4 μL 5× FastPfu buffer, 2 μL 2.5 mM dNTPs, 0.8 μL of each primer (5 μM), 0.4 μL FastPfu DNA polymerase, and 10 ng template DNA, with ddH_2_O added to volume. Thermal cycling parameters comprised: 95 °C for 3 min; 27 cycles of 95 °C (30 s), 55 °C (30 s), 72 °C (45 s); final extension at 72 °C for 10 min; hold at 4 °C. All samples were amplified in triplicate. The PCR product was extracted from 2% agarose gel and purified. Then quantified using Synergy HTX (Biotek, USA). Purified amplicons were pooled in equimolar amounts and paired-end sequenced on an Illumina NextSeq 2000 PE300 platform (Illumina, San Diego,USA) according to the standard protocols by Majorbio Bio-Pharm Technology Co. Ltd. (Shanghai, China). The raw sequencing reads were deposited into the NCBI Sequence Read Archive (SRA) database with the accession number PRJNA1259773.

### 2.10 Untargeted metabolomics analysis

#### 2.10.1 HPLC-MS analysis

##### 2.10.1.1 Bone marrow sample collection

For untargeted metabolomic analysis of murine bone marrow, mice were euthanized and the right femur was carefully dissected. Both ends of the bone were trimmed to expose the intramedullary cavity. A 23-gauge needle was then inserted, and the bone marrow cavity was thoroughly flushed with 1 mL of phosphate-buffered saline (PBS) by repeated irrigation until the entire marrow content was eluted. The resulting bone marrow suspension was collected for subsequent metabolomic profiling.

##### 2.10.1.2 Unconventional samples

Take all the bone marrow samples for freeze-drying. A 400 μL solution (Methanol: Water = 7:3, V/V) containing internal standard was added into the sample, and vortexed for 3 min. The sample was subjected to 10 min of sonication in an ice bath, followed by 1 min of vortexing and a 30 min incubation at −20 °C. The sample was then centrifuged at 12,000 rpm for 10 min (4 °C). And the sediment was removed, then centrifuged the supernatant at 12,000 rpm for 3 min (4 °C). A 200 μL aliquots of supernatant were transferred for LC-MS analysis.

##### 2.10.1.3 HPLC conditions

All samples were for two LC/MS methods. One aliquot was analyzed using positive ion conditions and was eluted from T3 column (Waters ACQUITY Premier HSS T3 Column 1.8 μm, 2.1 mm * 100 mm) using 0.1% formic acid in water as solvent A and 0.1% formic acid in acetonitrile as solvent B in the following gradient: 5%–20% in 2 min, increased to 60% in the following 3 min, increased to 99% in 1 min and held for 1.5 min, then come back to 5% mobile phase B within 0.1 min, held for 2.4 min. The analytical conditions were as follows, column temperature, 40 °C; flow rate, 0.4 mL/min; injection volume, 4 μL; Another aliquot was using negative ion conditions and was the same as the elution gradient of positive mode.

##### 2.10.1.4 MS conditions (AB)

Software information-dependent acquisition (IDA) mode was implemented for data acquisition using Analyst TF 1.7.1. (Sciex, Concord, ON, Canada). The source parameters were set as follows: ion source gas 1 (GAS1), 50 psi; ion source gas 2 (GAS2), 50 psi; curtain gas (CUR), 25 psi; temperature (TEM), 550 °C; declustering potential (DP), 60 V, or−60 V in positive or negative modes, respectively; and ion spray Voltage floating (ISVF), 5000 V or −4000 V in positive or negative modes, respectively. The TOF MS scan parameters were set as follows: mass range, 50–1,000 Da; accumulation time, 200 ms; and dynamic background subtract, on. The product ion scan parameters were set as follows: mass range, 25–1,000 Da; accumulation time, 40 ms; collision energy, 30 or−30 V in positive or negative modes, respectively; collision energy spread, 15; resolution, UNIT; charge state, 1 to 1; intensity, 100 cps; exclude isotopes within 4 Da; mass tolerance, 50 ppm; maximum number of candidate ions to monitor per cycle, 18.

#### 2.10.2 Data analysis

##### 2.10.2.1 PCA

Unsupervised PCA (principal component analysis) was performed by statistics function prcomp within R (www.r-project.org). The data was unit variance scaled before unsupervised PCA.

##### 2.10.2.2 Hierarchical cluster analysis and pearson correlation coefficients

The HCA (hierarchical cluster analysis) results of samples and metabolites were presented as heatmaps with dendrograms, while pearson correlation coefficients (PCC) between samples were calculated by the corfunction in R and presented as only heatmaps. Both HCA and PCC were carried out by R package Complex-Heatmap. For HCA, normalized signal intensities of metabolites (unit variance scaling) are visualized as a color spectrum.

##### 2.10.2.3 Differential metabolites selected

For two-group analysis, differential metabolites were determined by VIP (VIP > 1) and P-value (P-value< 0.05, Student’s t-test). Using the R package MetaboAnalystR, we performed OPLS-DA to obtain VIP scores and generated associated score plots and permutation plots. The data was log transform (log2) and mean centering before OPLS-DA. In order to avoid overfitting, a permutation test (200 permutations) was performed.

##### 2.10.2.4 KEGG annotation and enrichment analysis

Identified metabolites were annotated using KEGG Compound database (http://www.kegg.jp/kegg/compound/), annotated metabolites were then mapped to KEGG Pathway database (http://www.kegg.jp/kegg/pathway.html).

### 2.11 Statistical analysis

All data are presented as the mean ± SEM and represent 6 or more samples. For statistical evaluation, we utilized Prism 10.1.2 (GraphPad), performing one-way ANOVA complemented by Dunnett’s *post hoc* tests for intergroup comparisons. P values ≤ 0.05 were considered to be significant.

## 3 Results

### 3.1 Body weight

As showed in Figure 1B, all experimental groups exhibited progressive body weight gain throughout the initial 8-day observation period post-intervention, with intergroup comparisons revealing no significant variations (p > 0.05). On the 12th and 15th day, the mice’s weight of MDL group decreased significantly compared with CTL group (P < 0.05), whereas the mice’s weight of in SXF(M) and SXF(H) groups were higher than that in the MDL group.

### 3.2 SXF increased peripheral platelet count in CIT mice

Platelet quantification serves as a critical clinical parameter for chemotherapy decision-making, primarily to mitigate thrombocytopenia-related risks in oncology patients. Peripheral blood platelet counts were measured at 3-day intervals to evaluate SXF’s effects on CIT. From day 1 to day 12, peripheral platelets of mice in all groups except the IL-11 group increased slowly. On 12th day, which was the 4th day after carboplatin injection, the platelet counts of mice in SXF(M) and SXF(H) groups were higher than that in the MDL group (P < 0.05, P < 0.01). IL-11 administration induced a rapid platelet count increase (days 1–9) preceding a decline initiated at day 12 (Figure 1C).

On 15th day, which was the 7th day after carboplatin injection, the platelet count of mice in all groups except CTL group dropped to the lowest point, whereas the platelet counts of mice in SXF(M) and SXF(H) groups were higher than that in the MDL group (P < 0.05, P < 0.001). Thereafter, peripheral platelet count of mice in each groups began to rise. The SXF-treated groups demonstrated significantly faster platelet recovery (10 days post-chemotherapy) compared to the MDL group (16 days post-chemotherapy) (Figure 1C).

### 3.3 The protective effect of SXF on the bone marrow microenvironment of CIT mice

In order to evaluate the protective effect of SXF on the bone marrow microenvironment, hematopoietic stem/progenitor cells, megakaryocytic progenitor cells, megakaryocytes, other nucleated cells, cytokine levels and metabolomics in mice bone marrow microenvironment were assessed on the 15th day, which was the 7th day after the carboplatin injection.

#### 3.3.1 SXF protected hematopoietic stem/progenitor cells in bone marrow against carboplatin

**FIGURE 2:**
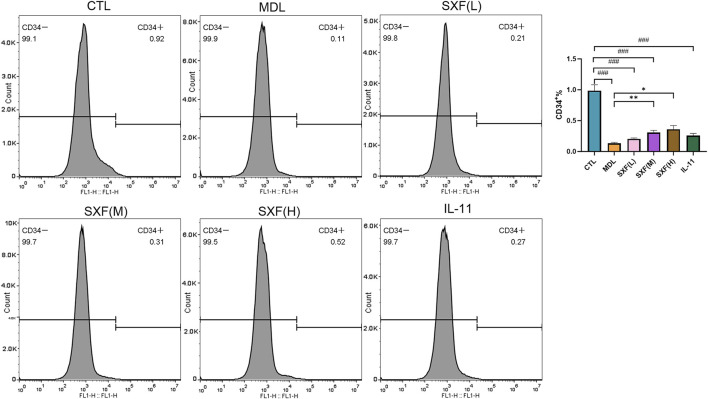
The expression of CD34 was detected by flow cytometry in BM cells of each group. The histogram indicates the percentage of CD34^+^ cells in each group. Data are reported as the mean ± SD (n = 10). ###p < 0.001, ##p < 0.01, #p < 0.05 versus the CTL group. ***p < 0.001, **p < 0.01, *p < 0.05 versus the MDL group.

Flow cytometric assessment of CD34 expression revealed significant depletion of bone marrow HSPCs in MDL mice versus CTL mice (P < 0.001). SXF administration produced concentration-responsive increases in CD34^+^ cell populations relative to untreated model animals (Figure 2).

#### 3.3.2 SXF protected megakaryocytic progenitor cells and megakaryocytes in bone marrow against carboplatin

**FIGURE 3:**
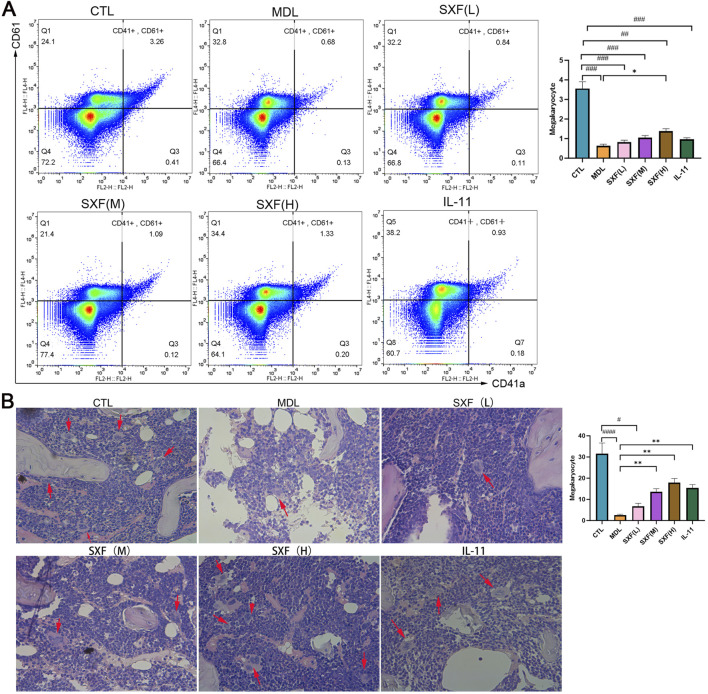
Therapeutic effects of SXF on bone marrow microenvironment. **(A)** The expression of CD41 and CD61 was detected by flow cytometry in BM cells of each group. The histogram indicates the percentage of CD41^+^CD61^+^ cells in each group. **(B)** Representative images of H&E staining of BM from all the groups. The red arrows indicate megakaryocytes. The histogram indicates the number of megakaryocytes in each group. Data are reported as the mean ± SD (n = 10). ^###^p < 0.001, ^##^p < 0.01, ^#^p < 0.05 versus the CTL group. ***p < 0.001, **p < 0.01, *p < 0.05 versus the MDL group.

Furthermore, we detected the expression of megakaryocytic-specific marker CD41a and CD61 by flow cytometry. The MDL group showed significantly lower percentages of CD41a^+^CD61^+^ cells in marrow cellularity versus CTL group by flow cytometric evaluation (P < 0.001). The proportion of CD41a^+^CD61^+^ cells were all significantly increased in SXF treated groups in a concentration-dependent manner compared with control group (Figure 3A). Comparative histopathology revealed significantly higher megakaryocyte density in SXF-treated cohorts relative to disease model controls (p < 0.05), exhibiting clear treatment concentration proportionality across 25%-50%–100% dosage gradients (Figure 3B).

#### 3.3.3 SXF restored the levels of TPO, IL-11, IL-6, IL-3 and restrained the levels of IL-1βand PDGFB

**FIGURE 4:**
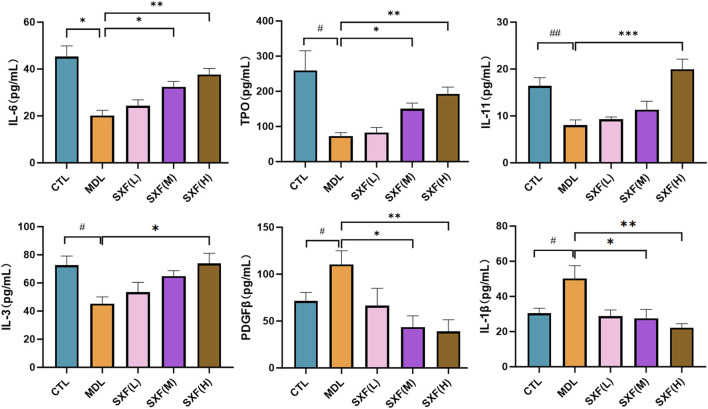
The cytokine levels of BM from all the groups. Data are reported as the mean ± SD (n = 10). ^###^p < 0.001, ^##^p < 0.01, ^#^p < 0.05 versus the CTL group. ***p < 0.001, **p < 0.01, *p < 0.05 versus the MDL group.

Cytokines play a crucial regulatory role in the hematopoiesis process in the bone marrow microenvironment. We detected the levels of cytokines related to platelet-production in the bone marrow. As shown in Figure 4, The MDL group exhibited significantly reduced concentrations of thrombopoietic cytokines (TPO, IL-11, IL-3, and IL-6) compared to controls (P < 0.05, P < 0.01) compared with CTL group. These cytokine levels were restored in SXF treated groups in a concentration-dependent manner compared with MDL group. Conversely, the IL-1β and PDGFB levels in SXF treated groups decreased in a dose-dependent manner with MDL group.

### 3.4 Intestinal microbiota

#### 3.4.1 Diversity analysis of intestinal microbiota

Fecal samples collected from CTL, MDL, and SXF(H)-treated mice were analyzed to establish baseline gut microbiota profiles. Our sequencing generated a total of 534,442 high-resolution reads at single-nucleotide level, averaging 59,382 reads per specimen. After removing the unqualified sequences, a total of 262,788 sequences each sample contained 26,622–32,298 sequences, resulting in 7,600 ASVs for further analysis.

**FIGURE 5:**
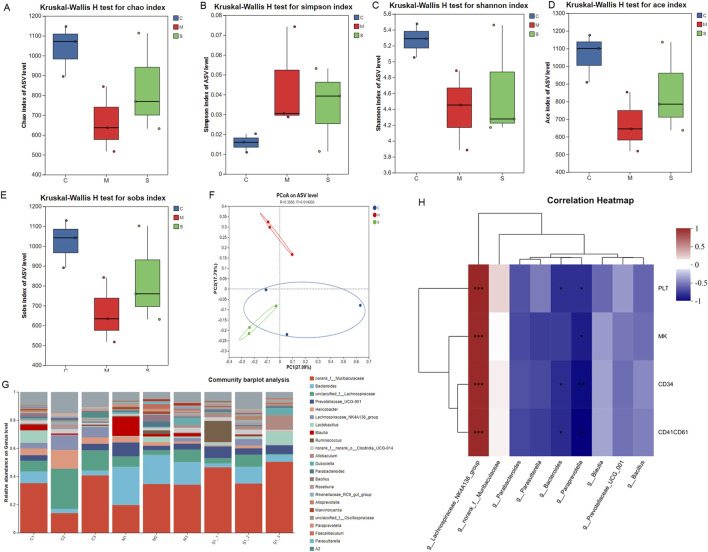
Therapeutic effects of SXF on gut microbiota. **(A–E)** Effects of SXF on alpha diversity of CIT mice, involving Chao, Simpson, Shannon, ace and sobs. **(F)** Effects of SXF on beta diversity of CIT mice, including PCoA analysis. **(G)** Relative abundance of the different bacterial phylum on Genus level. C, M, S respectively represent CTL, MDL and SXF(H) groups. **(H)** The correlation between key hematopoietic function indicators and gut microbiota. The X and Y-axes represent gut microbiota and hematopoietic function indicators, respectively. PLT, MK, CD34, CD41CD61 respectively represent platelet counts, megakaryocytes, CD34^+^ cell populations and CD41a^+^CD61^+^ cell populations. Correlation coefficients (R values) and corresponding P values were calculated. The R values are depicted using a color gradient in the plot, with statistically significant correlations (P < 0.05, 0.01, 0.001) indicated by asterisks (*, **, ***).

Alpha diversity indices, including Chao1, Sobs, and Shannon, were employed to assess intrasample species richness and evenness. Larger alpha diversity values are associated with higher microbial diversity. Compared to CTL and SXF(H) groups, MDL-treated mice exhibited significantly reduced alpha diversity indices (observed Chao1 index, p < 0.05; Sobs index, p < 0.05 and Shannon index, p < 0.01) (Figures 5A–E).

#### 3.4.2 Changes in the composition of the intestinal microbiota

A two-dimensional scatter plot visually represented the β-diversity patterns among the CTL, MDL, and SXF(H) groups. Beta diversity analysis revealed closer clustering between CTL and SXF(H) groups compared to CTL-MDL pairs, suggesting greater similarity in microbial community structure (Figure 5F). At the phylum level (Figure 5G), the relative abundance of *Bacteroides*, *Prevotellaceae_UCG-001*, *Blautia, Dubosiella*, *Parabacteroides*, *Bacillus* and *Paraprevotella* in MDL group was higher than that of the other groups. Taxonomic profiling identified significant dysbiosis in MDL mice, showing inverse abundance patterns for key genera - suppressed *Lachnospiraceae_NK4A136* (P < 0.05) alongside enriched *Paraprevotella* and *Bacteroides* (both P < 0.05) compared to controls. However, the SXF(H) group showed a dramatically reduced relative abundance of *Paraprevotella*, *Bacteroides* but an increased relative abundance of *Lachnospiraceae_NK4A136* compared with that in the model group (P < 0.05).

#### 3.4.3 Correlation between key hematopoietic function indicators and gut microbiota

Additionally, correlation analysis was performed to evaluate the relationships between key hematopoietic function indicators (platelet counts, megakaryocytes, CD34^+^ cell populations and CD41a^+^CD61^+^ cell populations) and differential microbial markers (*Lachnospiraceae_NK4A136*, *Paraprevotella*, *Bacteroides*). As shown in Figure 5H, spearman correlation analysis revealed that the abundance of *Lachnospiraceae_NK4A136* was positively correlated with all hematopoietic function indicators (P < 0.001). In contrast, the abundances of *Paraprevotella* and *Bacteroides* were negatively correlated with hematopoietic function markers (P < 0.05, P < 0.01). The results confirmed that the regulatory effect of SXF on gut microbiota is closely related to the improvement of hematopoietic function, as detailed in the correlation heatmap.

### 3.5 SXF affects bone marrow metabolites in CIT mice

**FIGURE 6:**
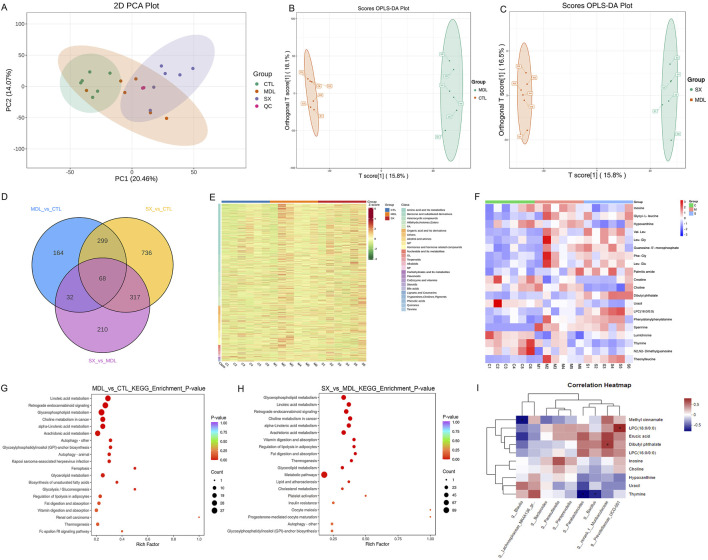
The effect of SXF on metabolomics of BM in CIT mice. **(A)** The PCA score plot. **(B)** OPLS-DA score plot of MDL group and CTL group. **(C)** OPLS-DA score plot of SXF(H) group and MDL group. **(D)** Venn analysis illustrates the unique and shared differential metabolites among three comparative groups: MDL versus CTL, SXF(H) versus CTL, and SXF(H) versus MDL. **(E,F)** Differential metabolite heat maps derived from BM. **(G,H)** Main metabolic pathway impact analysis between MDL_vs_CTL group and SXF(H)_vs_MDL group. **(I)** The correlation between BM metabolites and gut microbiota in SXF(H) group and MDL group mice. The X and Y-axes represent gut microbiota and bone marrow metabolites, respectively. Correlation coefficients (R values) and corresponding P values were calculated. The R values are depicted using a color gradient in the plot, with statistically significant correlations (P < 0.05) indicated by asterisks (*). The legend on the right shows the color scale for the R values. Hierarchical clustering was performed for gut microbiota and bone marrow metabolites, as shown on the left and top sides of the figure, respectively.

To elucidate the potential metabolic mechanisms underlying SXF’s therapeutic effects on CIT, we conducted untargeted metabolomic profiling of bone marrow samples using HPLC-MS/MS technology, following the observed gut microbiota modifications. PCA and OPLS-DA analysis showed a divergence among the samples in the positive and negative modes (Figures 6A–C). The Venn diagram showed that a total of 68 metabolites are shared among the three groups of metabolites (Figure 6D). Inosine, hypoxanthine, choline, uracil, and thymine were significantly downregulated while dibutyl phthalate, LPC(16:0/0:0), LPC(18:0/0:0), erucic acid and methyl cinnamate were upregulated in the CIT model group after treatment with SXF(H) (Table 2; Figures 6E,F). Based on the results of differential metabolites, KEGG pathway enrichment analysis was performed. Comparative pathway analysis revealed significant dysregulation (p < 0.01/0.05) across four signaling cascades when comparing MDL_VS_CTL and SXF(H)_VS_MDL conditions, with parallel metabolomic profiling identifying concomitant perturbations in: (i) linoleic acid metabolism, (ii) glycerophospholipid turnover, (iii) endocannabinoid retrograde signaling, and (iv) oncogenic choline metabolic flux (Figures 6G,H). Therefore, the lipid metabolism pathway was affected most significantly after treatment with SXF(H). Platelet activation was also among the enriched pathways.

**TABLE 2:** Differential metabolites (SX_VS_MDL).

Index	Metabolites	Type
MW0159913	Inosine	Down
MW0159863	Hypoxanthine	Down
MEDP0125	Choline	Down
MEDL01942	Dibutyl phthalate	Up
MW0169988	Uracil	Down
MW0054524	LPC(16:0/0:0)	Up
MW0126805	Thymine	Down
MW0012998	LPC(18:0/0:0)	Up
MEDL02002	Erucic acid	Up
MW0138954	Methyl cinnamate	Up

### 3.6 Correlation analysis between intestinal microbiota abundance and bone marrow metabolites

The relationship between genus-level intestinal microbiota and bone marrow metabolites was assessed via Spearman’s correlation analysis (r > 0.5). The affected intestinal microbiota were significantly correlated with different bone marrow metabolites. *Blautia* was negatively correlated with the upregulated metabolites in the comparison results between the SXF(H) group and MDL group. Spearman correlation analysis revealed significant positive associations between *Parabacteroides*, *Prevotellaceae_UCG-001*, *Bacillus*, and unclassified *Muribaculaceae* with upregulated metabolites, while showing inverse correlations with downregulated metabolites in SXF(H) versus MDL group comparisons (Figure 6I). These findings delineate a dual-phase therapeutic mechanism whereby SXF alleviates CIT through restoration of intestinal microbial ecology coupled with profound reprogramming of marrow metabolic networks.

## 4 Discussion

The incidence of chemotherapy-induced thrombocytopenia (CIT) is rising annually, paralleling the increasing global burden of cancer (Sathishkumar et al., 2022; Wan et al., 2023; Rahib et al., 2021). The mechanisms underlying CIT primarily encompass three aspects: decreased platelet production (Machlus et al., 2014), increased platelet destruction (Beg et al., 2008), and abnormal platelet distribution (Zorzi et al., 2007). In this study, we established CIT mouse model by injecting carboplatin through the tail vein (Akiyama et al., 1997), primarily observing the suppression of hematopoietic stem cell and megakaryocyte progenitor cell proliferation, a reduction in megakaryocyte production in the bone marrow microenvironment, and a decrease in both megakaryocyte generation and platelet release. The study provides compelling evidence that SXF intervention can ameliorate chemotherapy-associated bone marrow suppression in CIT models. SXF exhibits specific protective effects on multiple hematopoietic cells, including HSCs, HPCs, megakaryocytic lineage cells, and other thrombopoietic precursors within the bone marrow niche. Additionally, SXF plays a role in the recovery of platelet-relevant hematopoietic cytokine levels and expedites the normalization of platelet counts. Therefore, the obtained results indicate that SXF alleviates the damage to the bone marrow microenvironment caused by carboplatin. However, the underlying mechanism remains to be explored in greater depth and detail.

Emerging evidence highlights the gut microbiota as a pivotal modulator of bone marrow hematopoiesis (Liu et al., 2024). The differentiation capacity of hematopoietic stem/progenitor cells is regulated by intestinal microbiota diversity and population dynamics (Fernandez et al., 2024), highlighting its substantial involvement in chemotherapy-associated pathologies (Liu et al., 2022). Our results demonstrated that carboplatin treatment altered the gut microbiome profile by: (a) promoting the growth of *Bacteroides* and *Paraprevotella*, while (b) suppressing *Lachnospiraceae_NK4A136*. Notably, the observed reduction in this beneficial bacterial group has been implicated as a potential mediator of chemotherapy-associated dysbiosis (Yan et al., 2023). SXF intervention normalized microbial composition, particularly restoring *Lachnospiraceae_NK4A136*, a known producer of short-chain fatty acids (SCFAs) that enhance intestinal barrier integrity and immune homeostasis (Cao et al., 2023). SCFAs (especially butyrate) serve as essential energy sources and regulatory factors for bone marrow hematopoietic stem cells (Xu et al., 2018; Adeniyi et al., 2023). We also performed functional prediction of the key genus-level gut microbiota using PICRUSt2, and pathway enrichment analysis of the correlated bone marrow metabolites via the KEGG database. The results (Supplementary Figure S4A) show that the SXF-regulated gut microbiota are mainly involved in metabolite biosynthesis and metabolic pathway regulation. Among them, the differential metabolites were enriched in Glycosphingolipid biosynthesis, and the pathway diagram of differential metabolites is shown in Supplementary Figure S4B. The correlated Ceramide synthases (CerS) and sphingomyelinases (SMase) enriched in pathways influence the maturation process of megakaryocytes by regulating sphingolipid metabolic pathways (de Jonckheere et al., 2023). This supplementary analysis complements the existing mechanistic discussion and enhances the persuasiveness of the proposed regulatory pathway. Spearman correlation analysis further revealed significant associations between specific microbiota and bone marrow metabolites, implying microbiota-mediated metabolic regulation of hematopoiesis.

Untargeted metabolomics revealed that SXF treatment effectively reversed carboplatin-mediated metabolic dysregulation in bone marrow, with most pronounced effects on linoleic acid metabolism and glycerophospholipid biosynthesis pathways. The upregulation of LPCs (e.g., LPC(16:0/0:0), LPC(18:0/0:0)) was notable, as these lipids are essential for cell membrane synthesis and can activate G protein-coupled receptors (e.g., G2A) to promote megakaryocyte maturation and platelet release (Machlus et al., 2014). Concurrently, reduced purine metabolites (e.g., inosine, hypoxanthine) may alleviate oxidative stress by suppressing ROS generation, thereby protecting HSC function (Zhang et al., 2023). These metabolic alterations were strongly associated with gut microbiota restructuring, demonstrated by inverse correlations between *Blautia* and LPCs, as well as positive associations between *Parabacteroides* and purine metabolites. Such findings underscore a hierarchical “microbiota-metabolite-bone marrow” regulatory network, implying microbiota-mediated metabolic regulation of hematopoiesis. These findings lend credence to the novel “gut-bone marrow axis” hypothesis, wherein intestinal microbial communities exert distant regulatory effects on bone marrow activity through metabolic-immune interactions.

However, it is important to acknowledge the limitations of the current study. While our correlation analysis strongly suggests a link between SXF-mediated microbiota modulation and hematopoietic recovery, direct causal evidence - such as that derived from germ-free or pseudo-germ-free mouse models or *in vitro* functional validation of specific metabolites - was not established in this work. The carboplatin-induced non-tumor-bearing CIT mouse model employed here is a classic preclinical model focusing on hematopoietic protection, but verification in tumor-bearing models is necessary to ensure SXF does not interfere with the anti-tumor efficacy of chemotherapy. We plan to address these critical questions in future studies by conducting germ-free animal experiments and evaluating the impact of SXF on the chemotherapeutic efficacy of carboplatin in tumor-bearing mice.

This study reveals that SXF alleviates CIT through a “microbiota-metabolism” regulatory axis, providing mechanistic insights into traditional Chinese medicine (TCM) for managing chemotherapy toxicity. Compared to conventional therapies (e.g., rhIL-11), SXF offers multi-target efficacy with reduced toxicity, making it suitable for long-term chemotherapy patients. Future efforts should prioritize the development of standardized SXF formulations and personalized microbiota modulation strategies (e.g., probiotic adjuvants) to refine CIT management. The proposed gut-bone marrow regulatory axis could potentially revolutionize therapeutic approaches for chemotherapy-associated hematological complications.

## 5 Conclusion

In summary, our findings establish SXF as an effective therapeutic intervention for carboplatin-associated thrombocytopenia in preclinical models. Our findings suggest a dual mechanistic framework: sustained marrow hematopoietic competence working in concert with adaptive reprogramming of both enteric microbiotal populations and their metabolic repertoire.

## Data Availability

The datasets presented in this study can be found in online repositories. The names of the repository/repositories and accession number(s) can be found below: https://www.ncbi.nlm.nih.gov/, PRJNA1259773.
