# Supplementary material for: Si xian formula (SXF) alleviates carboplatin-induced bone marrow microenvironment damage and promotes thrombocytopenia recovery by regulating gut microbiota and bone marrow metabolites: a correlative study
Source: Front Pharmacol. 2026 Apr 10;17:1689477. doi: 10.3389/fphar.2026.1689477 (PMC13105887; doi:10.3389/fphar.2026.1689477)
Supplement: Supplementary file 1 [file Supplementaryfile1.docx]

Supplementary Material

# Determination of Astragaloside IV Content in SXF by HPLC

Chromatographic Conditions ：A octadecylsilane-bonded silica gel column was used as the stationary phase; the mobile phase consisted of acetonitrile: water (30:70); detection was carried out using an evaporative light scattering detector. The number of theoretical plates calculated for the astragaloside IV peak should not be less than 4,000.

Astragaloside IV reference standard was accurately weighed and dissolved in methanol to prepare a solution containing 0.5 mg per 1 ml. 9 g SXF was accurately weighed, to which 40 ml of methanol was added. The mixture was subjected to ultrasonic treatment for 30 minutes, then taken out and allowed to cool. The solvent in the extract was recovered and concentrated to dryness. The residue was gently heated with 10 ml of water to dissolve, followed by shaking with water-saturated n-butanol for extraction. This extraction process was repeated four times, each time using 40 ml of n-butanol. The n-butanol layers were combined and thoroughly washed twice with ammonia solution, each time using 40 ml. The ammonia layers were discarded, and the n-butanol layer was evaporated to dryness. The residue was dissolved in methanol and transferred to a 5 ml volumetric flask. Methanol was added to the mark, and the solution was mixed well. Accurately inject 5 μl and 10 μl of the reference solution and 10 μl of the test solution into the liquid chromatograph. SXF was sampled in triplicate and identical measurements were performed. Determine the content using a logarithmic equation based on the two-point external standard method.

The HPLC chromatogram is shown in Figure S1. The content of Astragaloside IV in SXF was calculated to be 39.27 mg per 100 g.

# Determination of Icariin Content in SXF by HPLC

# Chromatographic Conditions ：An octadecylsilane-bonded silica gel column was used as the stationary phase, with a mobile phase consisting of acetonitrile-water (27:73). The detection wavelength was set at 270 nm, and the number of theoretical plates for the icariin peak should not be less than 1,500.

Icariin reference standard was accurately weighed and dissolved in methanol to prepare a solution containing 0.1 mg per 1 ml. Approximately 2 g of SXF was accurately weighed and placed in a stoppered conical flask. Then, 20 ml of dilute ethanol was precisely added, and the total weight was recorded. The mixture was subjected to ultrasonic treatment for 30 minutes. After completion, the flask was taken out and allowed to cool to room temperature. The weight was measured again, and the weight loss was compensated by adding dilute ethanol. The solution was mixed well, filtered, and the subsequent filtrate was collected for use. SXF was sampled in triplicate and identical measurements were performed. Accurately inject 10 μl each of the reference solution and the tests solution into the liquid chromatograph, perform the measurement, and obtain the result.

The HPLC chromatogram is shown in Figure S2. The content of Icariin in SXF was calculated to be 6.84 mg per 100 g.

# Detection under the third LC condition

Chromatographic Conditions ：It was performed on a column packed with octadecylsilane-bonded silica gel; the mobile phase consisted of acetonitrile–0.4% acetic acid aqueous solution (15:85); the detection wavelength was set at 330 nm.

Approximately 2 g of SXF was accurately weighed and placed in a stoppered conical flask. Then, 20 ml of dilute ethanol was precisely added, and sonicate for 30 minutes. Remove the flask, allow it to cool to room temperature, weigh it again, and adjust the weight to the original with dilute ethanol. Shake well, filter, and use the successive filtrate as the test solution. Accurately pipet 10 μL of the test solution, inject it into the high-performance liquid chromatograph, perform the measurement, and obtain the result. The HPLC chromatogram is shown in Figure S3.

# Functional prediction of the key genus-level gut microbiota

we performed functional prediction of the key genus-level gut microbiota using PICRUSt2, and pathway enrichment analysis of the correlated bone marrow metabolites via the KEGG database. The results (Figure S4A) show that the SXF-regulated gut microbiota are mainly involved in metabolite biosynthesis and metabolic pathway regulation. Among them, the differential metabolites were enriched in Glycosphingolipid biosynthesis, and the pathway diagram of differential metabolites is shown in Figure S4B.

# Supplementary Figures and Tables

## Supplementary Figures


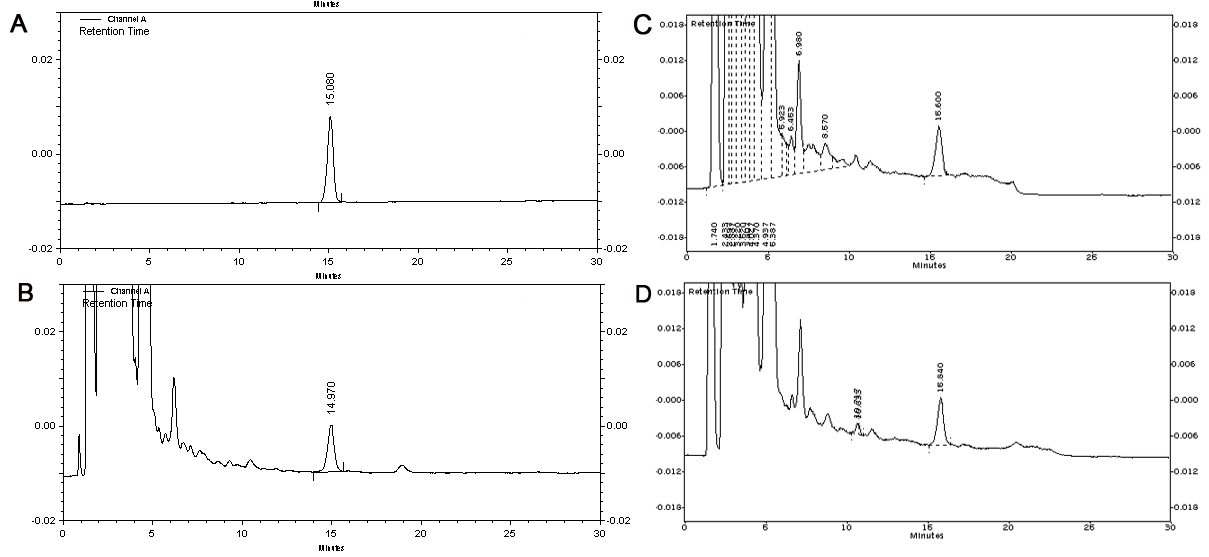


Figure S1 HPLC chromatograms of Astragaloside IV Content in SXF by HPLC

(A) HPLC chromatogram of astragaloside IV reference standard. (B-D) HPLC chromatograms of three distinct SXF extracts.


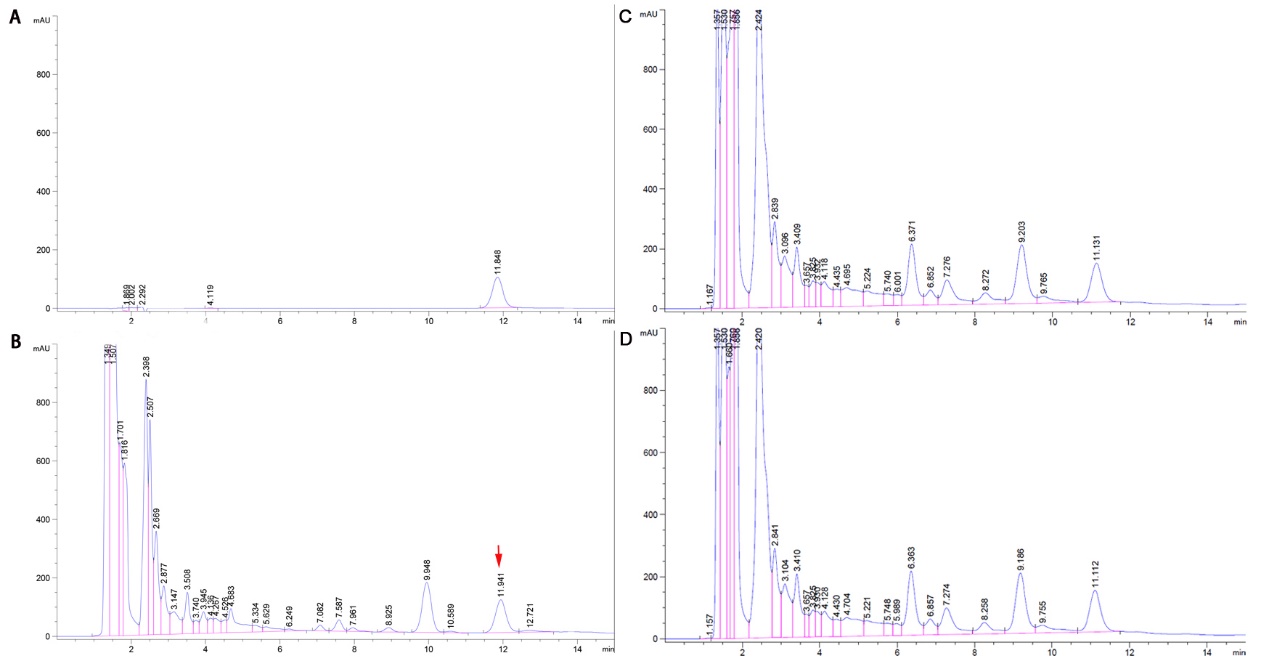


Figure S2 HPLC chromatograms of Icariin Content in SXF by HPLC

(A) HPLC chromatogram of Icariin reference standard. (B-D) HPLC chromatograms of three distinct SXF extracts.


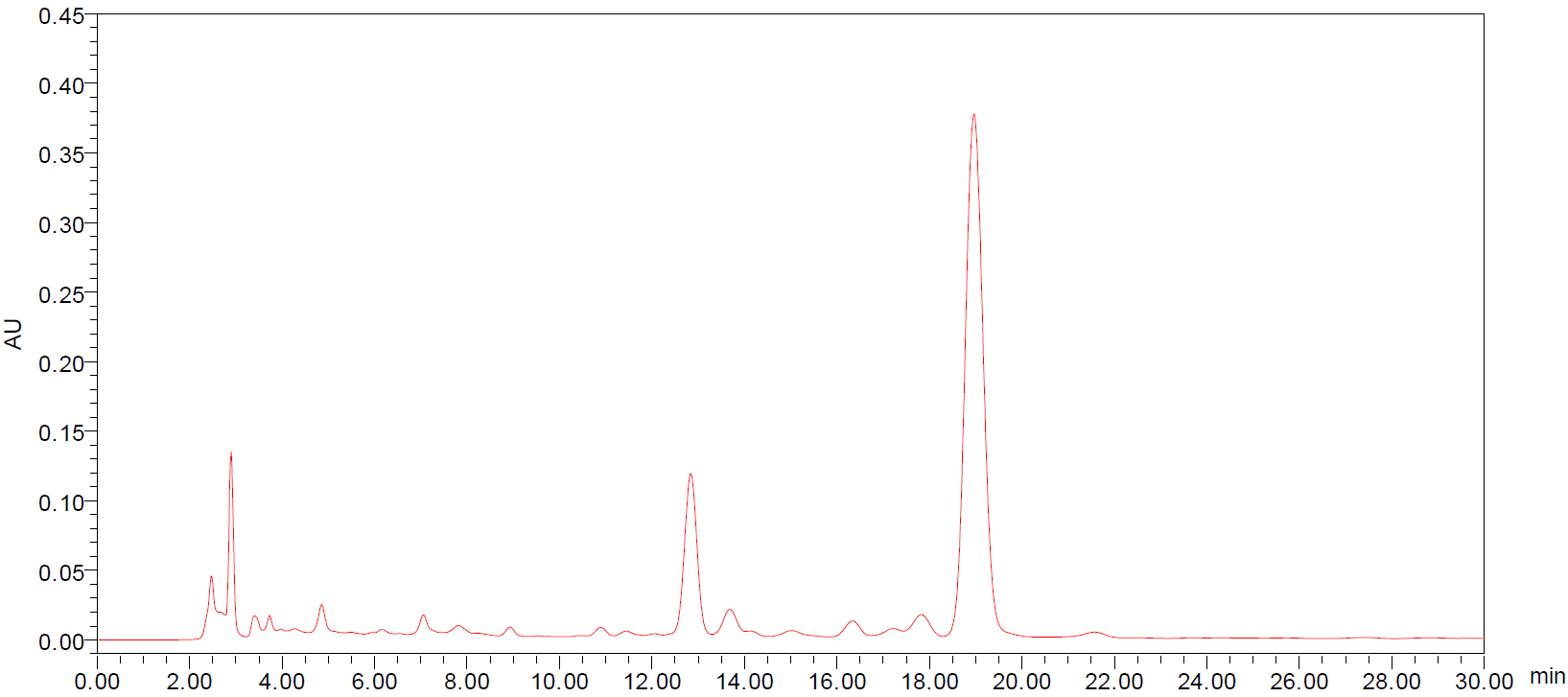


Figure S3 Functional prediction of the key genus-level gut microbiota


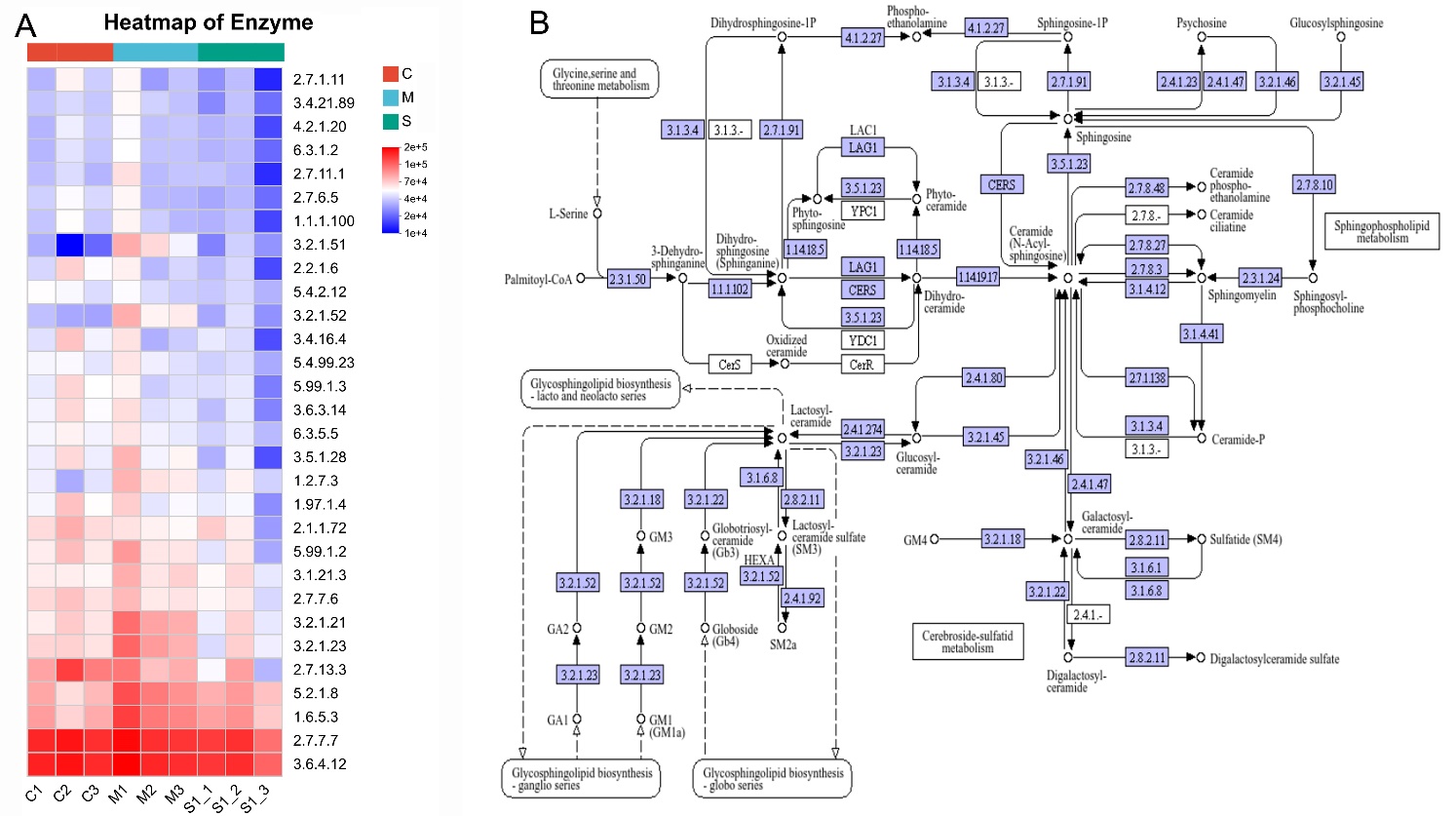


Figure S4 HPLC chromatograms of SXF by HPLC
